# USP47-mediated deubiquitination and stabilization of YAP contributes to the progression of colorectal cancer

**DOI:** 10.1007/s13238-019-00674-w

**Published:** 2019-11-20

**Authors:** Beiqing Pan, Yi Yang, Jian Li, Yu Wang, Chuantao Fang, Fa-Xing Yu, Yanhui Xu

**Affiliations:** 1 Fudan University Shanghai Cancer Center, Institutes of Biomedical Sciences, State Key Laboratory of Genetic Engineering, Key Laboratory of Medical Epigenetics and Metabolism, Shanghai Medical College of Fudan University, Shanghai, 200032 China; 2 Children’s Hospital and Institutes of Biomedical Sciences, Key Laboratory of Medical Epigenetics and Metabolism, Fudan University, Shanghai, 200032 China; 3 Key Laboratory of Molecular Medicine, Ministry of Education, Department of Systems Biology for Medicine, School of Basic Medical Sciences, Shanghai Medical College of Fudan University, Shanghai, 200032 China; 4 Collaborative Innovation Center of Genetics and Development, School of Life Sciences, Fudan University, Shanghai, 200433 China; 5 CAS Center for Excellence in Molecular Cell Science, Chinese Academy of Sciences, Shanghai, 200031 China

**Dear Editor,**

The Hippo tumor suppressor pathway plays an important role in development and tumorigenesis. Yes-associated protein (YAP) is a major effector of the Hippo pathway regulating the expression of genes involved in cell proliferation, cell death, and cell differentiation (Pan, 2010; Johnson and Halder, 2014; Piccolo et al., 2014; Yu et al., 2015). In intestine, YAP is mainly expressed in leucine rich repeat containing G protein-coupled receptor 5 (LGR5) marked stem cells, and is required for regeneration following tissue damage and the development of colon cancer in mice (Cai et al., 2010; Barry et al., 2013; Wang et al., 2017). Moreover, in colorectal cancer (CRC) specimens, elevated YAP expression and activity is frequently observed (Steinhardt et al., 2008). It has been shown that high YAP expression is associated with cancer metastasis, poor prognosis, resistance to chemotherapy, and greater possibility of relapse (Johnson and Halder, 2014; Yu et al., 2015). However, the molecular mechanism underlying elevated YAP protein expression in CRC and other cancers remains poorly defined.

The ubiquitin-proteasome system (UPS) plays a critical role in tumorigenesis (Popovic et al., 2014). Dysregulated expression of E3 ligases or deubiquitinating enzymes (DUB) is frequently observed in human cancers, including CRC. The protein stability of YAP is mainly regulated by phosphorylation and ubiquitination, and the latter is carried out by beta-transducin repeat containing E3 ubiquitin protein ligase (β-TRCP) (Zhao et al., 2010). However, the DUB responsible for YAP deubiquitination and stabilization in CRC is currently unknown.

It has been shown previously that ubiquitin specific peptidase 7 (USP7, a DUB) interacts with β-TRCP (Peschiaroli et al., 2010). We speculated that β-TRCP and USP47 may work together to fine-tune the ubiquitination and protein stability of YAP. To verify this hypothesis, we first tested the interaction between USP47 and YAP. In co-immunoprecipitation (Co-IP) assays, recombinant USP47 could pull-down YAP and *vice versa*, suggesting that USP47 and YAP physically interact with each other (Fig. 1A). The inter-molecular interaction was mediated by the peptidase domain (C19C) and coiled coil (CC) domains in USP47 and N-terminal domain in YAP (Fig. S1). In an *in vitro* deubiquitination assay, addition of USP47 led to deubiquitination of YAP in a dose dependent manner (Fig. S2). Consistently, overexpression of USP47 resulted in a decrease of ubiquitination of ectopic or endogenous YAP in HEK293T cells (Fig. 1B and 1C). Moreover, knockdown of USP47 in HEK293T cells increased YAP ubiquitination (Fig. 1D and 1E). Taken together, these results indicate that YAP physically interacts with USP47, and USP47 serves as a DUB for YAP.

**Figure 1:**
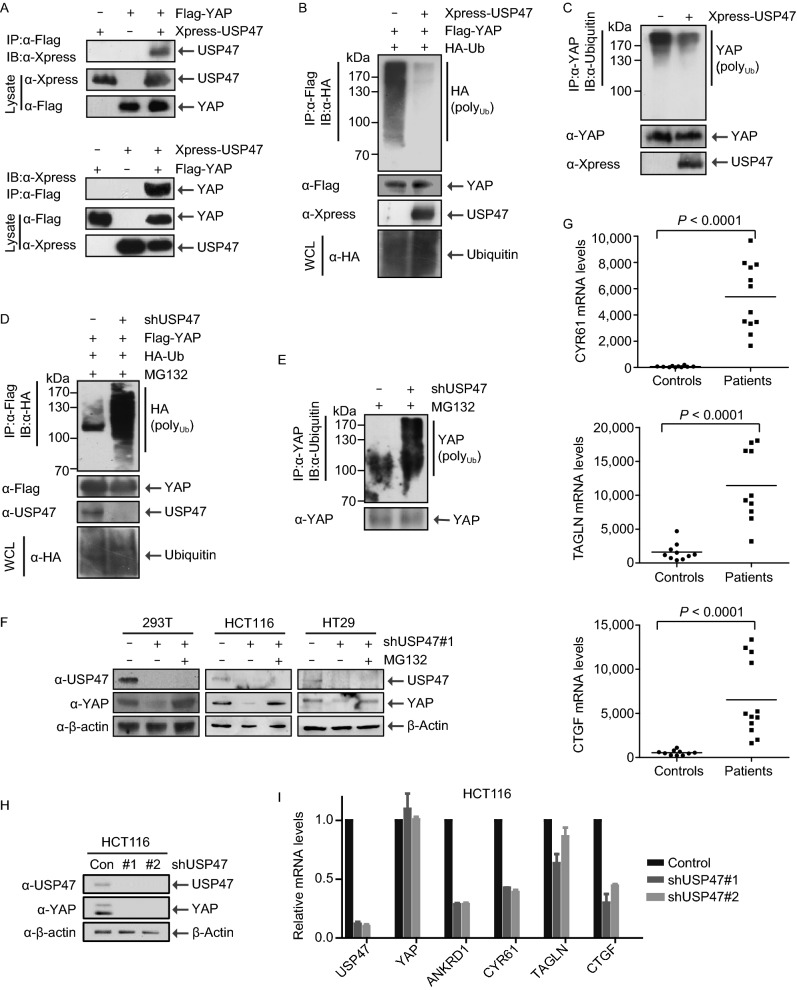
**USP47 serves as a DUB for YAP and induces expression of YAP target genes in CRC cells**. (A) YAP interacts with USP47. Flag-YAP and Xpress-USP47 expression plasmids were co-transfected into HEK293T cells, the interaction between YAP and USP47 was determined by immunoprecipitation with α-Flag beads (top) or α-Xpress beads (bottom) followed by immunoblotting with α-Xpress or α-Flag antibody. One percent of whole cell lysates were loaded as input control. (B) USP47 deubiquitinates YAP in cells. Xpress-USP47, Flag-YAP and HA-Ub expression plasmids were co-transfected into HEK293T cells. The ubiquitination of precipitated YAP was analyzed by immunoblotting with anti-HA antibody. (C) USP47 deubiquitinates endogenous YAP. Xpress-USP47 expression plasmids were transfected into HEK293T cells, the ubiquitination of precipitated endogenous YAP was analyzed by immunoblotting with anti-ubiquitin antibody. (D) Knockdown of USP47 promotes YAP ubiquitination. Flag-YAP and HA-Ub expression plasmids were co-transfected into HEK293T-shCtr or HEK293T-shUSP47 cells, and cells were treated with MG132 (20 µmol/L). The ubiquitination of precipitated YAP was analyzed by immunoblotting with anti-HA antibody. (E) Knockdown of USP47 promotes endogenous YAP ubiquitination. Endogenous YAP was immunoprecipitated from HEK293T-shCtr or HEK293T-shUSP47 cells pretreated with MG132 (20 µmol/L). The ubiquitination of YAP was analyzed by immunoblotting with anti-ubiquitin antibody. (F) Knockdown of USP47 promotes YAP degradation. HEK293T-shUSP47, HCT116-shUSP47 and HT29-shUSP47 cell lines and control cells were treated with or without MG132 (20 µmol/L). The expression levels of USP47, YAP and actin were determined. (G) mRNA expression analysis of YAP target genes from GEO dataset GDS2609 of colon mucosae from early onset CRC patients and healthy controls. (H and I) Knockdown of USP47 dramatically decreased YAP protein level and its target genes expression. The protein expression levels of USP47, YAP and actin were determined with antibodies indicated (H). The relative mRNA levels of USP47, YAP and YAP target genes were quantified using RT-qPCR, *n* = 3 (I)

Ubiquitination often is coupled with protein destabilization via proteasomal degradation. In the presence of cycloheximide (CHX, an inhibitor of protein translation), overexpression of USP47 notably delayed the protein turnover of YAP in HEK293T cells (Fig. S3). On the other hand, in HEK293T, HCT116, and HT29 cells, knockdown of USP47 induced YAP degradation, and this effect was rescued by inhibition of proteasome (by MG132) or ectopic expression of shRNA resistant USP47 (Figs. 1F and S4). Thus, USP47 is critical in regulating protein stability of YAP.

We retrieved gene expression omnibus (GEO) dataset GDS2609 which contains mRNA expression data of colon mucosae from early onset CRC patients and healthy controls, and analyzed the mRNA expression patterns of *USP47*, *YAP*, and YAP target genes. *YAP* mRNA levels in CRC samples and healthy controls are similar, however, the mRNA levels of *USP47* and representative YAP target genes, such as *CYR61*, *TAGLN*, and *CTGF*, are increased in CRC patients’ mucosae (Figs. 1G and S5). Hence, *USP47* mRNA level is elevated at the early stage of CRC, and may promote the expression of YAP target genes. Consistently, in HCT116 cells, knockdown of USP47 has no effect on *YAP* mRNA level but dramatically decreased YAP protein levels and the expression of YAP target genes (Fig. 1H and 1I). Collectively, these data suggest that USP47 promotes YAP stability and transcriptional activity through a post-transcriptional mechanism.

We also tested the effect of OTU domain-containing ubiquitin aldehyde-binding protein 2 (OTUB2) and OTU deubiquitinase 1 (OTUD1), two recently reported DUBs for YAP in mammary cells (Yao et al., 2018; Zhang et al., 2019). In HCT116 cells, knockdown of OTUB2 resulted in YAP destabilization (Fig. S6). Hence, USP47 and OTUB2 may play overlapping function in regulating YAP stability in colon cancer cells.

To explore pathological functions of USP47, we analyzed the expression of *USP47* in a GEO dataset (GSE32323) containing mRNA levels of 17 paired CRC tumors and adjacent normal tissues. The mRNA levels of *USP47* were about 20% higher in CRC tumors than that in paired normal tissues (Fig. S7A). In another GEO dataset (GSE17537) containing survival data of CRC patients, high *USP47* expression was correlated with shorter overall survival (Fig. S7B). Moreover, we assessed protein levels of USP47 and YAP in 90 CRC tumor samples with paired normal tissues on a tissue microarray. As indicated by immunohistochemistry (IHC) staining, the protein levels of USP47 were higher in tumor tissues, especially in tumors with bigger size (>15 mm^3^) (Fig. 2A and 2B). Similarly, YAP protein levels were also up-regulated in tumor tissues (Fig. 2A). Interestingly, the USP47 protein level was positively correlated with YAP protein level in the CRC patient samples (Fig. 2C), and a strong co-expression of USP47 and YAP were frequently overserved in high grades (III and IV) CRC specimens (Table S1). Together, these results demonstrate that the protein levels of both USP47 and YAP are elevated in CRC, and concurrent high USP47 and YAP expression is an indicator of aggressive tumors.

**Figure 2:**
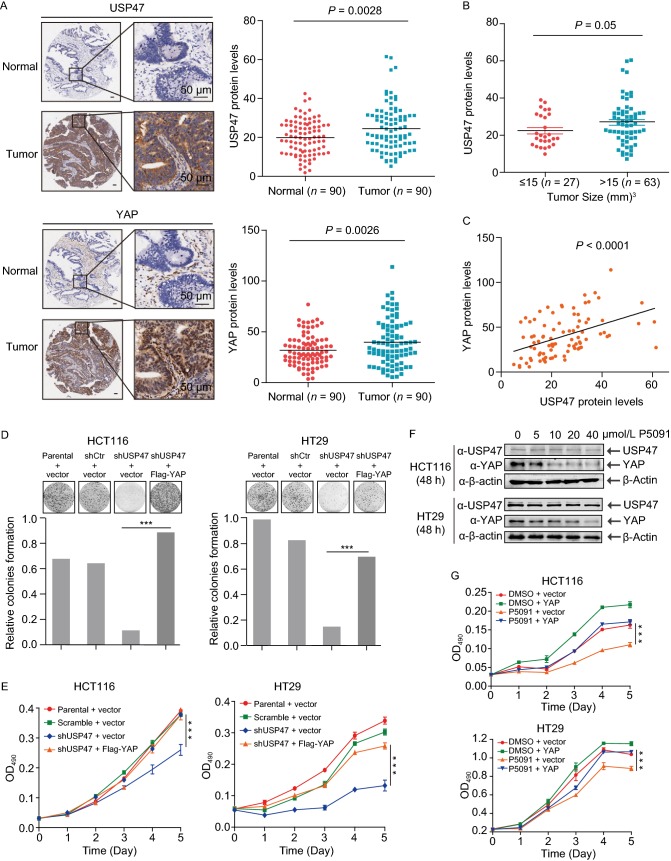
**USP47-regulated stabilization of YAP is involved in the development of CRC**. (A) Elevated USP47 and YAP protein expression in CRC specimens. USP47 and YAP expression were determined by IHC. (B) USP47 protein levels were higher in in tumors bigger than 15 mm^3^. (C) YAP and USP47 protein expression in CRC specimens were positively correlated. (D and E) Overexpression of YAP rescued USP47 knockdown-induced inhibition of cell proliferation. (F) USP47 inhibitor P5091 decreases YAP protein expressions in HCT116 and HT29 cells. USP47, YAP and actin protein expressions were determined following P5091 treatment. (G) USP47 inhibitor P5091 suppressed HCT116 and HT29 cells growth and the inhibition was reversed by YAP overexpression. The data are presented as the means ± SD from triplicate experiments. ***P < 0.001, Student’s *t* test

Given that USP47 is overexpressed in CRC patients, we reasoned that inhibition of USP47 may suppress CRC cell growth. To explore this possibility, we first performed colony formation assay (anchorage-independent growth) and growth curve assay in CRC cell lines with or without USP47 knockdown. In both HT29 and HCT116 cells, USP47 knockdown led to a decreased number of colonies and slower cell proliferation (Fig. S8). To test if down-regulation of YAP played a role in cell growth retardation in USP47 knockdown cells, we ectopically expressed YAP in USP47 knockdown cells. The colony formation and cell proliferation were rescued by ectopic expression of YAP, suggesting that YAP is involved in the growth-promoting function of USP47 (Fig. 2D and 2E). To further investigate the role of USP47 in CRC cell proliferation and growth, we treated HCT116 and HT29 cells with P5091, a small molecule inhibitor for USP7 and USP47 (Chauhan et al., 2012; An et al., 2017). P5091 treatment enhanced YAP ubiquitination and reduced YAP protein level in a dose-dependent manner (Figs. 2F and S9). Moreover, P5901 suppressed proliferation of HCT116 and HT29 cells, and the inhibition was also reversed by YAP overexpression (Fig. 2G). The effect of P5091 on YAP was mainly dependent on USP47, as knockdown of USP7 had no effect on YAP protein levels (Fig. S10). These data suggest that inhibition of USP47, by knockdown or chemical inhibition, leads to destabilization of YAP, and low YAP protein expression in turn attenuates CRC cell proliferation.

 In summary, we uncovered an USP47-YAP signaling axis in regulating the development of CRC. Both USP47 and YAP are highly expressed in CRCs, and their high expression is associated with aggressiveness of CRC. Mechanistically, USP47 functioned as a DUB for YAP, and elevated USP47 expression lead to stabilization of YAP oncoprotein. Moreover, USP47 inhibition by RNA interference or a chemical inhibitor can repress CRC cell proliferation by decreasing YAP expression. Our study hence shed lights on molecular mechanisms underlying YAP activation in CRC and possibly other cancers, and demonstrated the proof-of-principle of using USP47 inhibitors for treating YAP-dependent cancers.

## Electronic supplementary material

Below is the link to the electronic supplementary material.

Supplementary material 1 (PDF 721 kb)
