# Supplementary material for: USP47-mediated deubiquitination and stabilization of YAP contributes to the progression of colorectal cancer
Source: Protein Cell. 2019 Nov 20;11(2):138–43. doi: 10.1007/s13238-019-00674-w (PMC6954888; doi:10.1007/s13238-019-00674-w)
Supplement: Supplementary file 1 — Supplementary material 1 (PDF 721 kb) [file 13238_2019_674_MOESM1_ESM.pdf]

SUPPLEMENTARY MATERIALS AND METHODS

USP47 protein purification

USP47 gene was cloned from cDNA of HEK293T cells and constructed into a pFastBac1 derivative encoding a His6 tag and a 3C protease cleavage site. Recombinant USP47 was expressed in Sf9 insect cells using the Bac-to-Bac baculoviral system (Invitrogen). USP47 protein was purified to homogeneity by Nickel Nitrilotriacetic Acid affinity chromatography followed by 3C protease cleavage, anion exchange and gel filtration chromatography.

In vitro YAP deubiquitination assay

Flag-YAP and HA-Ubiquitin expressing plasmids were co-transfected into HEK293T cells for 48 hr, Ubiquitinated YAP was then immunoprecipitated using α-Flag beads and followed by *in vitro* deubiquitination assay, in which 1 μg or 3 μg purified USP47 proteins were incubated with Ub-YAP in 30 μl reaction volume containing 50 mM Tris-HCl (pH 8.0), 150 mM $NaCl_2$, 5% glycerol, 1mM EDTA (pH 8.0), and 2 mM DTT. After incubation in 30 ℃ for 2 hr, the beads were washed three times with corresponding washing buffer, and the immobilized proteins were dissolved in 1 X SDS running buffer and subjected to SDS-PAGE. Ubiquitinated proteins were analyzed by immunoblotting with YAP or HA antibody.

Cell Culture and Transfection

HEK293T cells and the human CRC cell lines HCT116 /HT29 were cultured in

DMEM medium (Gibco) or RPMI 1640 (Sangon) medium separately supplemented with 10% fetal bovine serum (Bioind), 2 mM L-glutamine(Sangon), penicillin (100 U/mL), and streptomycin (100 mg/mL) in 5% CO2 at 37 ℃. Cells were transfected with siRNA and plasmid vectors using Lipofectamine 6000 (Beyotime).

Stable Cells

The ORF of YAP was cloned into lentiviral vector pLVX-IRES-neo for stable cells establishment. The USP47 short hairpin sequences (#1): 5’-GAAGCATTGCATGCATTTATT -3’ and the scrambled sequence: 5’-TTCTCCGAACGTGTCACGTTT-3’were cloned into pLKO.1-TRC vector for stable cells establishment. DNA constructs (pLKO.1-TRC or pLVX-IRES-neo) were co-transfected with lentiviral packaging plasmids psPAX2 and pMD2.G into HEK293T cells. Lentiviral particles were collected twice separately at 48 hr and 72 hr after transfection to infect target cells supplemented with 10 μg/ml final concentration polybrene (Sigma). After 24 hr, stables clones were selected with 400 μg/ml G418 (Sangon) for vector pLVX-IRES-neo and 4 μg/ml puromycin (Sigma) for vector pLKO.1-TRC.

Colony Formation Assay and Growth Curve

The proliferation potential of cells was assessed by plating 2000/4000/8000 cells separately in 6-well plates. After 10 days of growth, cells were fixed with methanol, then stained with crystal violet (Sigma) for 30 mins at room temperature and the

number of colonies was counted. For the growth curve assay, 2000 cells were plated in 96-well plates with four replicates. CellTiter 96® AQueous One solution cell proliferation assay Kit (Promega) was used to measure the cell growth. The original medium was removed and 100 μl empty medium mixed with 20 μl agent was added in per well. After 1 hr of incubation, the absorbance was measured at OD490. The absorbance of the initial time point was measured after 6 hr of seeding cells to normalize the following results. Then the absorbance was measured every 24 hours.

Immunoblotting

Whole cell extracts were prepared with RIPA buffer (Vazyme Biotech) supplemented a mix of proteasome inhibitors. Protein concentration was detected by Bradford protein assay kit (Beyotime). Then cell lysates were separated by SDS-PAGE and the proteins were transferred to NC membrane (Millipore). Membranes were blocked with 5% skim milk dissolved in TBST. Immunoblotting was performed with primary antibodies against USP47 (Proteintech), YAP/TAZ(Cell Signaling Technology), USP7(Santa Cruz), p53(Santa Cruz) , HA (Santa Cruz), Xpress (Santa Cruz), Flag (Abmart), ubiquitin (Cell Signaling Technology), and β-actin (Abmart). β-actin was used as loading control. Molecular weight marker, ECL substrates, and image acquisition equipment (5200S) were from Tanon Science & Technology Co., Ltd.

Statistical analysis

All statistical analyses were performed using SPSS program (SPSS Inc, Chicago, IL).

Experimental results were evaluated statistically using the Student *t* test, the Student *t*' test, the Pearson chi-square test, and the ANOVA test. $P < 0.05$ was considered statistically significant. Datasets GSE23232 and GSE17537 were downloaded from GEO database and relevant information was obtained by GEO2R.

RNA isolation and Real-time PCR

Total RNA was prepared with Trizol reagent (Invitrogen) and the cDNA was synthesized using M-MLV Reverse Transcriptase (TaKaRa). Quantitative real-time PCR (qRT-PCR) was performed using a 7500 detection system (Applied Biosystems) and Go Taq qPCR Master Mix (Promega). The relative mRNA levels of target samples to that of control samples were calculated according to $2^{-\Delta\Delta Ct}$ method, in which the difference in Ct values (Δ Ct) between the target gene and the reference gene internal control (*GAPDH*) was calculated for normalization and the ΔCt of the different samples was compared directly ($\Delta\Delta$Ct).

Inhibitors

The human CRC cell lines (HCT116 and HT29) were incubated with USP47/USP7 dual inhibitor P5091 (SelleckChem) at the indicated concentrations and time. To determine the mechanism in which USP47 regulates the protein level of YAP, cells were treated with 20μM MG132 (Sigma) or 100μg/ml CHX (INALCO) to inhibit proteasome or protein synthesis separately. Vehicle-treated cells were used as controls.

Immunohistochemistry

Tissue microarrays containing 90 colon tumors and the corresponding normal colonic mucosa were from by Shanghai Outdo Biotech Co., Ltd (Cat No. FM-S4006-1 Vision1.0/Lot No. HCol-Ade180CS-01). Tissue microarrays or adjacent tissues were incubated with USP47 antibody (Santa Cruz), or YAP antibody (Cell Signaling Technology). Immunohistochemistry was performed using EnVision Kit from DAKO. The staining intensity was scored using ImageJ software.

siRNA and Oligos

The oligonucleotides were synthesized chemically by Sangon Biotech (Shanghai, China). The shRNA target sequences used were as follows:

USP7 (#1):5'-CCTGGATTTGTGGTTACGTTA

USP7 (#2):5'-CGTGGTGTCAAGGTGTACTAA

USP47(#1):5'-GCAGCTTTCAAACAACATTTA

USP47(#2):5'-GAAGCATTGCATGCATTTATT

USP47(#3): 5'-GCAGTCAACATGATTTCATTG (3'UTR)

USP47(#4): 5'-GCAGGTCACATGCAACAAATT (3'UTR)

OTUD1(#1): 5'-GGTGTCTACCATGATTCATTA

OTUD1(#2): 5'-CTGAATGTGAATATCCATTTA

OTUB2(#1): 5'-TCCGTTTACCTGCTCTATAAA

OTUB2(#2): 5'-CCTATGTGTCACTGGATTATT

Scramble：5’-TTCTCCGAACGTGTCACGTTT-3’

Real-time PCR primers used were：

GAPDH： 5’-ACAACTTTGGTATCGTGGAAGG

5’-GCCATCACGCCACAGTTTC

YAP: 5’-TAGCCCTGCGTAGCCAGTTA

5’-TCATGCTTAGTCCACTGTCTGT

USP47: 5’-AACCACAGATGTTACAAGGAGC

5’-AAGATATGTGTCGATTCGCCAG

CYR61： 5’-CTCGCCTTAGTCGTCACCC

5’-CGCCGAAGTTGCATTCCAG

CTGF： 5’-AAAAGTGCATCCGTACTCCCA

5’-CCGTCGGTACATACTCCACAG

TAGLN： 5’-CCGTGGAGATCCCAACTGG

5’-CCATCTGAAGGCCAATGACAT

ANKRD1： 5’-AGTAGAGGAACTGGTCACTGG

5’-TGTTTCTCGCTTTTCCACTGTT

OTUD1: 5’-TACATCGCCGACCATCTCG

5’-TCTCCAGCCTCCCTCCAGT

OTUB2: 5’-TGCTCACGTCGGCCTTCATC

5’-GTGGTGGTTCAGGGCGGTAT

SUPPLEMENTARY FIGURES

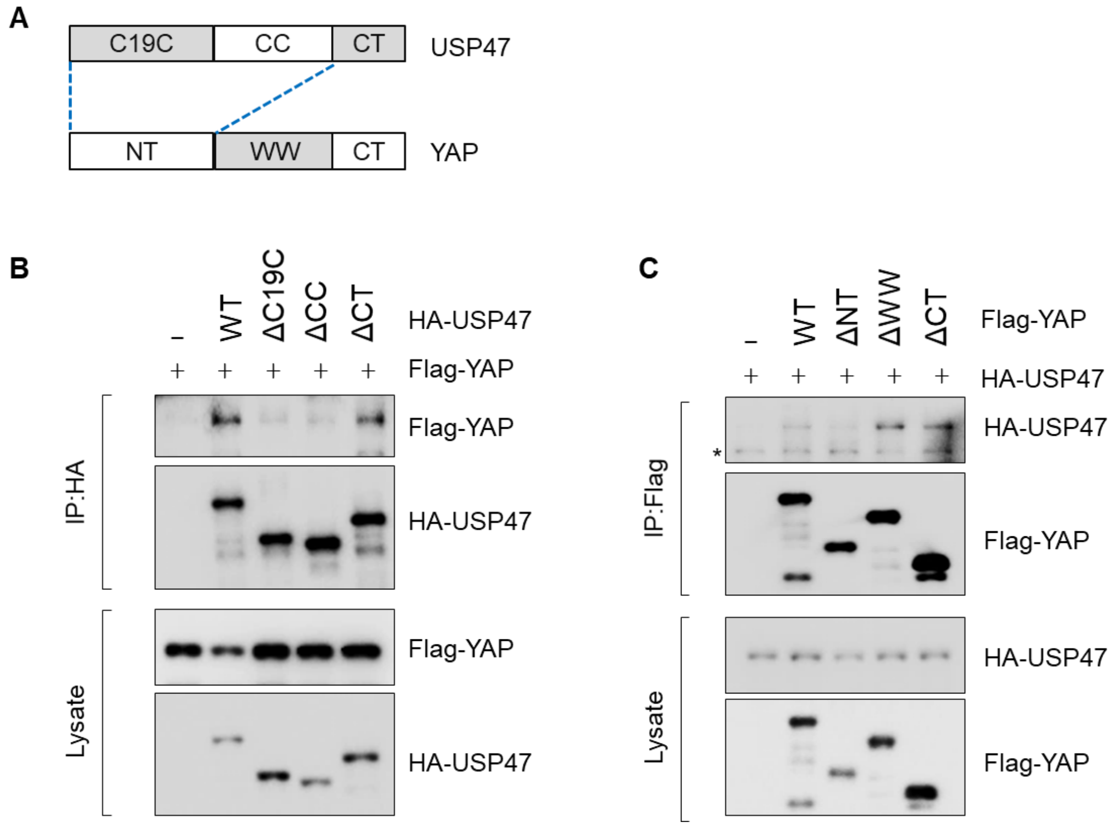

Figure S1. Protein domains involved in intermolecular interaction between USP47 and YAP.

(A) Protein domains of USP47 and YAP, related deletion mutants were used in co-immunoprecipitations (Co-IP) in B and C;

(B) C19C and CC domains of USP47 is required for interaction with YAP. HA-tagged USP47 deletion mutants were co-transfected with Flag-tagged YAP into HEK293T cells, and cell lysates were used for Co-IP with HA antibody;

(C) N-terminal (NT) of YAP is required for interaction with USP47. Fla-tagged YAP were co-transfected with HA-tagged USP47 into HEK293T cells, and cell lysates were used for Co-IP with Flag antibody.

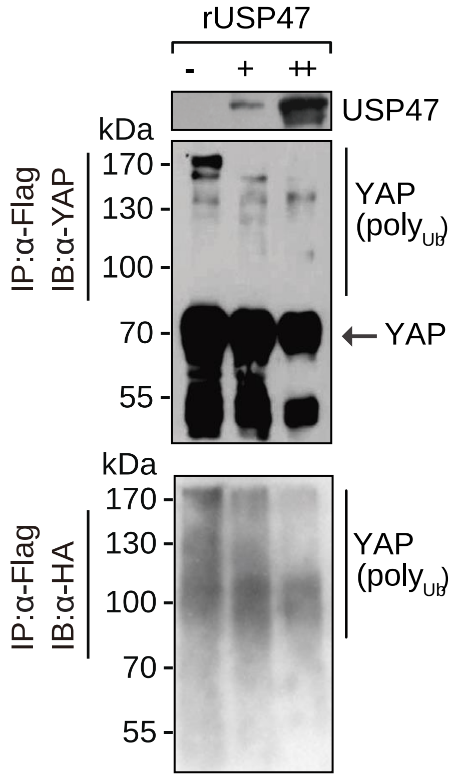

Figure S2. USP47 deubiquitinates YAP *in vitro*.

Flag-YAP and HA-Ub were overexpressed in HEK293T cells. Flag-YAP was immunoprecipitated with α-Flag beads and then incubated with recombinant USP47 protein. The ubiquitination of YAP was analyzed by immunoblotting with anti-YAP or anti-HA antibody.

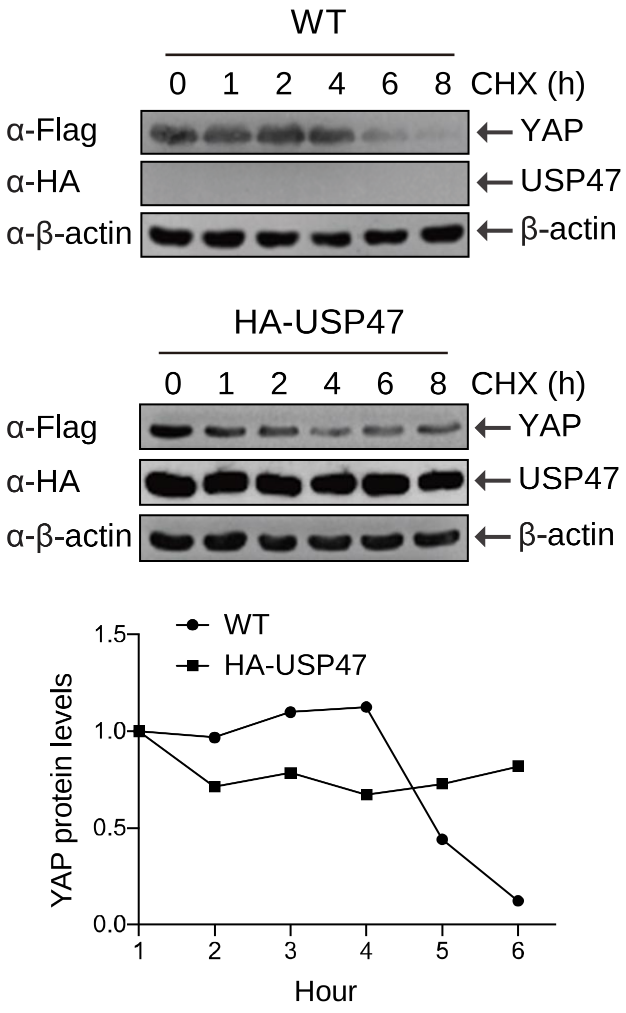

Figure S3. YAP is more stable in USP47-overexpressing cells.

Flag-YAP and HA-USP47 expression or control plasmids were co-transfected into HEK293T cells, and cells were treated with CHX (100 μg/ml) and harvested at indicated time points. The expression levels of YAP, USP47 and actin were determined by immunoblotting (top) and quantified (bottom).

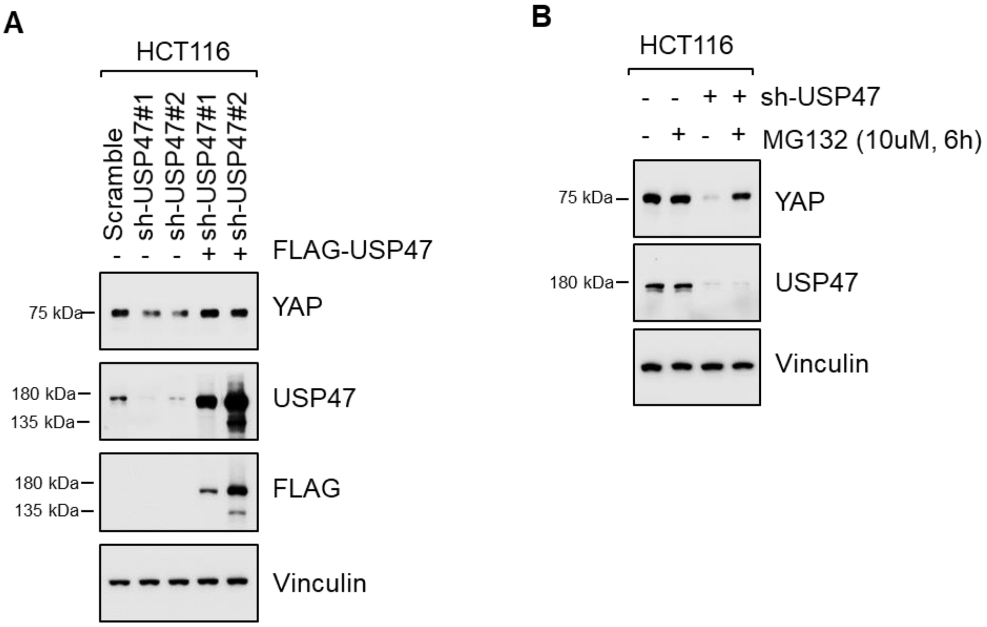

Figure S4. Effects of USP47 knockdown and re-expression on YAP protein levels.

(A) USP47 is critical in regulating YAP stability. YAP protein levels were decreased in HCT116 cells transfected with shRNAs targeting 3'UTR region of USP47, and ectopic expression of USP47 rescued YAP expression;

(B) USP47 inhibits proteasomal degradation of YAP. The effect of USP47 knockdown was largely blocked in the presence of proteasome inhibitor MG132.

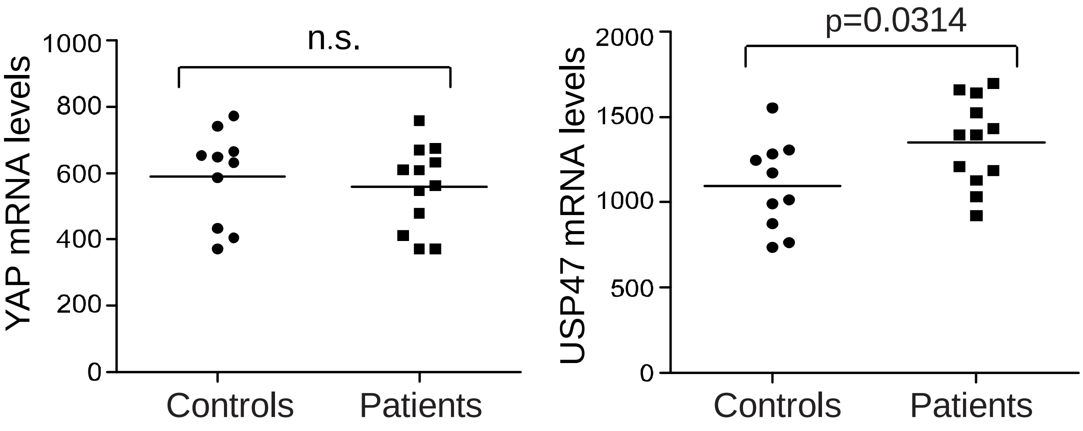

Figure S5. Upregulated USP47 expression in early onset CRC specimens.

mRNA expression analysis of YAP and USP47 in GEO dataset GDS2609 of colon mucosae from early onset CRC patients and healthy controls.

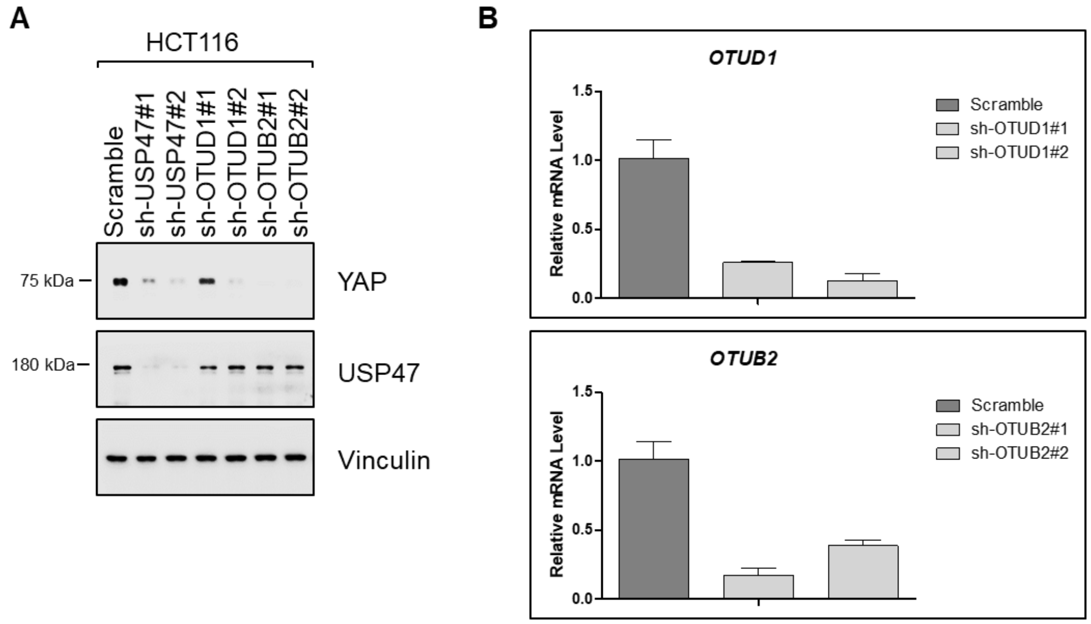

Figure S6. Effect of different DUB on YAP protein levels.

(A) Effect of different DUB on YAP protein levels. USP47, OTUD1, or OTUB2 was knocked down in HCT116 cells, and YAP protein levels were examined by immunoblotting;

(B) The down regulation of OTUD1 or OTUB2 mRNA levels by corresponding shRNA in HCT116 cells.

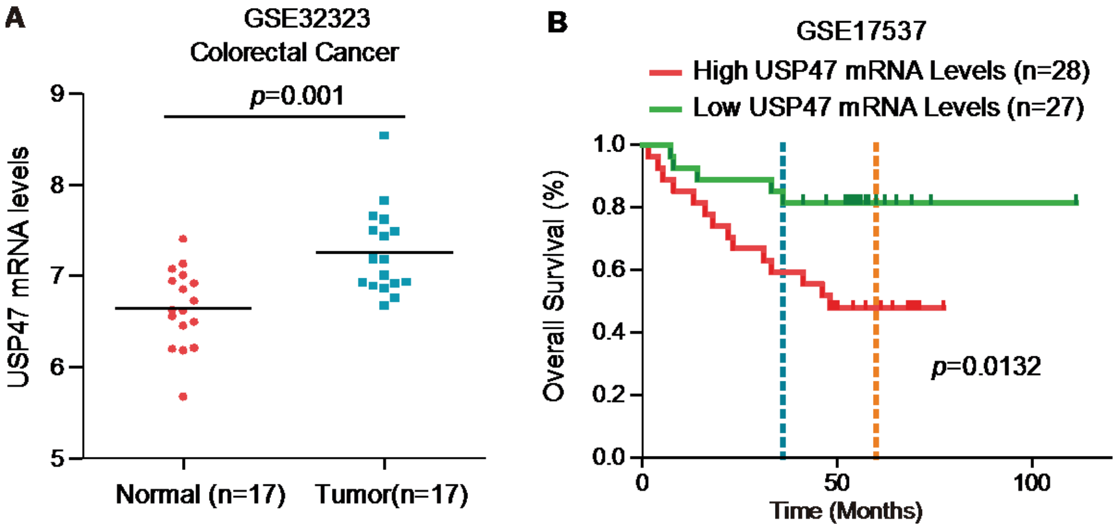

Figure S7. Elevated USP47 expression in CRCs predicts a poor prognosis.

(A) mRNA expression analysis of USP47 from GEO dataset (GSE32323) containing mRNA expression data of 17 paired CRC tumors and adjacent normal tissues.

(B) CRC patients (with high or low USP47 mRNA expression) overall survival analysis from GEO dataset (GSE17537).

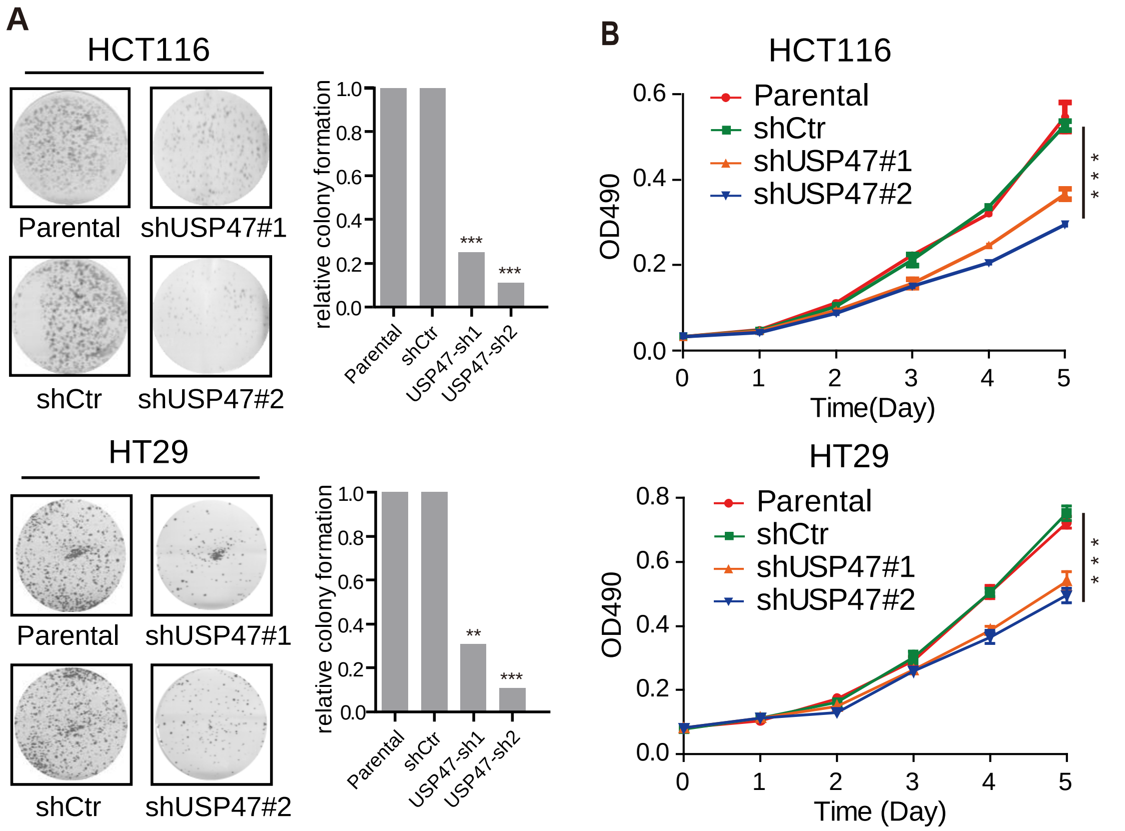

Figure S8. USP47 is involved in CRC cell proliferation.

USP47 knockdown inhibited proliferation of HCT116 and HT29 cells in colony formation assay (A) and growth curve assay (B).

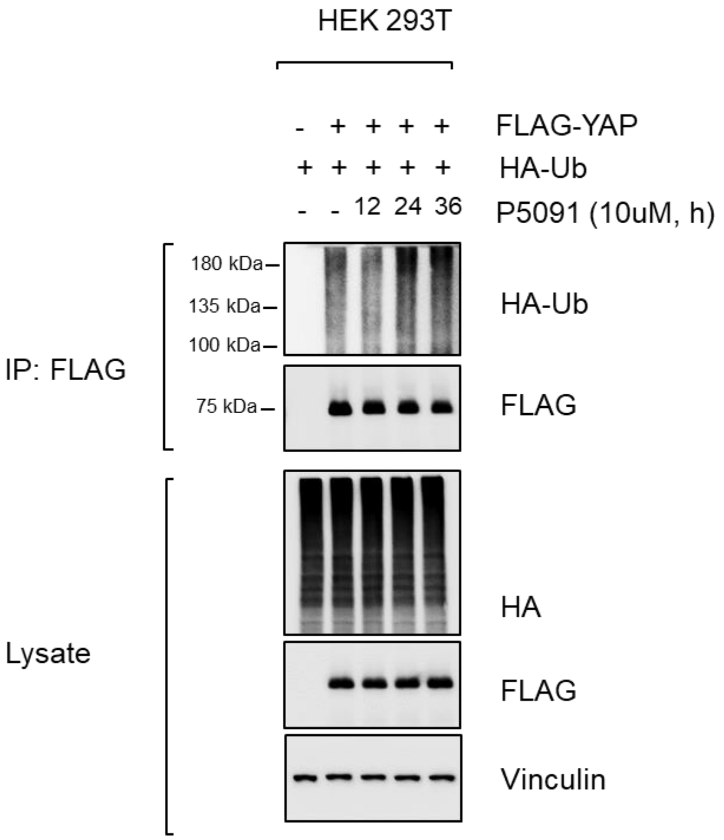

Figure S9. Effect of USP7/USP47 inhibitor on YAP ubiquitination.
HEK293T cells were transfected with Flag-tagged YAP or HA-tagged Ub, and treated with or without P5091 for indicated time. Flag-YAP was immunoprecipitated and probed with HA antibody. The HA-Ub signaling was increased in cells treated with P5091 for 24 or 36 hrs.

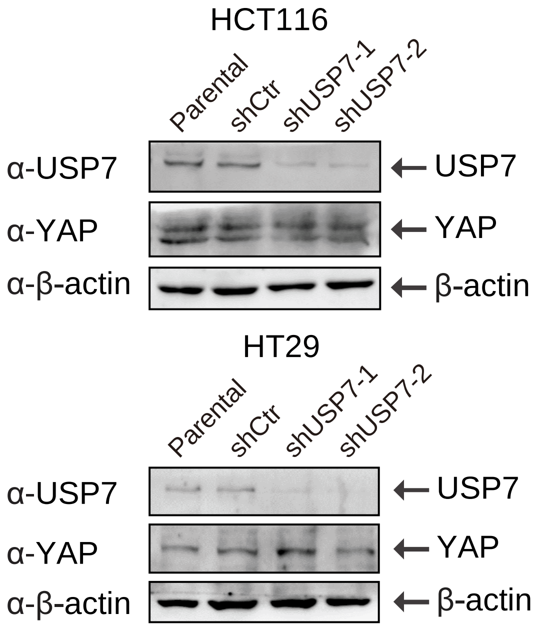

Figure S10. Knockdown of USP7 has no effect on YAP protein expression.
USP7, YAP and β-actin protein expressions were determined in control or USP7 knockdown HCT116 and HT29 cells.

SUPPLEMENTARY TABLE

Table S1. The expression levels of USP47 or YAP in CRCs.

Tumors		USP47+		YAP+		USP47+/YAP+	
Grade	n	n	%	n	%	n	%
I	17	7	41	8	47	3	18
II	23	14	61	5	22	4	17
III	35	17	49	17	49	13	37
IV	15	9	60	5	33	4	27
Total	90	47	52	35	39	24	27
